# Fasting blood glucose-to-glycated hemoglobin ratio and functional outcomes in patients with ischemic stroke following endovascular treatment—a meta-analysis

**DOI:** 10.3389/fneur.2025.1692103

**Published:** 2025-12-04

**Authors:** Chen Hong, Xin Wang, Hui Chen, Ying Sun

**Affiliations:** Department of Neurosurgery, The Second Affiliated Hospital of Harbin Medical University, Harbin, China

**Keywords:** glucose-to-glycated hemoglobin ratio, ischemic stroke, functional outcome, stress hyperglycemia, meta-analysis

## Abstract

**Background:**

Stress hyperglycemia is common in acute ischemic stroke (IS) and has been linked to adverse outcomes. The fasting glucose-to-glycated hemoglobin (HbA1c) ratio (GAR) has been proposed as a simple marker reflecting stress hyperglycemia relative to chronic glycemic status. Still, its prognostic value in patients undergoing endovascular treatment (EVT) remains unclear. This meta-analysis aimed to evaluate the association between GAR and 90d-day functional outcomes in these patients.

**Methods:**

PubMed, Embase, Web of Science, Wanfang, and Chinese National Knowledge Infrastructure (CNKI) were systematically searched through May 29, 2025. Studies reporting the association between GAR and poor functional outcome, defined as a modified Rankin Scale (mRS) score of 3–6 at 90 days, were included. Odds ratios (ORs) with 95% confidence intervals (CIs) were pooled using a random-effects model accounting for heterogeneity.

**Results:**

Twelve datasets from 10 retrospective cohort studies involving 3,878 patients were analyzed. The pooled analysis showed that a high GAR was significantly associated with an increased risk of poor functional outcome at 90 days after EVT (OR: 2.94, 95% CI: 2.22–3.88, *p* < 0.001; I^2^ = 14%). Meta-regression indicated that the proportion of diabetic patients showed a trend toward explaining the observed heterogeneity (coefficient = −0.0088, *p* = 0.08; I^2^ residual = 0%), whereas other factors showed no significant influence. Subgroup analyses yielded consistent results across age, sex, diabetes status, National Institutes of Health Stroke Scale (NIHSS), GAR cutoffs, and study quality (*p* for subgroup difference all > 0.05). The certainty of evidence for the primary outcome was rated as moderate according to the GRADE framework, mainly due to the retrospective design.

**Conclusion:**

High GAR may independently predict poor 90-day functional outcomes after EVT in patients with IS, supporting its potential prognostic value in clinical practice.

## Introduction

Ischemic stroke (IS) remains one of the leading causes of mortality and long-term disability worldwide, accounting for the majority of all stroke cases and imposing a significant global health burden (1, 2). Despite advances in prevention and acute care, the incidence of IS continues to rise due to aging populations and the growing prevalence of vascular risk factors such as hypertension, diabetes, and atrial fibrillation (3). Endovascular treatment (EVT) has revolutionized IS management, especially for large-vessel occlusions, by achieving higher reperfusion rates and better functional outcomes than intravenous thrombolysis alone (4, 5). However, even with technically successful recanalization, many patients fail to regain functional independence, suggesting that factors beyond vessel reopening influence prognosis (6). Identifying reliable predictors of functional outcome after EVT is therefore crucial for early risk stratification, individualized decision-making, and optimization of post-procedural care (7).

Stress hyperglycemia, reflecting acute metabolic stress during critical illness, is common in IS and has been linked to worse clinical outcomes through mechanisms including oxidative stress, endothelial dysfunction, inflammation, and impaired fibrinolysis (8, 9). To better characterize stress hyperglycemia, indices that adjust acute glucose levels for chronic glycemic status using glycated hemoglobin (HbA1c) have been proposed (10). These include admission glucose/HbA1c, admission glucose/estimated chronic glucose, and fasting glucose/HbA1c ratios (11). All three reflect acute-on-chronic glycemic imbalance, but the fasting glucose-to-HbA1c ratio—also termed the glucose-to-HbA1c ratio (GAR)—is less influenced by prandial fluctuations and may better capture true stress hyperglycemia (11). Emerging evidence suggests that GAR provides superior predictive value for mortality and poor functional outcomes compared with other indices in patients with IS receiving intravenous thrombolysis (12, 13). Nevertheless, studies evaluating GAR in IS patients treated with EVT remain limited. To the best of our knowledge, 10 retrospective cohort studies including 3,878 patients have examined GAR in this setting, with sample sizes ranging from 79 to 691 (14–23). Most reported that higher GAR was associated with an increased risk of poor 90-day functional outcome, although the strength of the association varied across studies (14–23). Accordingly, we conducted a meta-analysis to comprehensively assess the association between GAR and 90-day functional outcomes in IS patients undergoing EVT and to explore potential sources of heterogeneity across studies.

## Methods

We conducted this systematic review and meta-analysis in accordance with the Preferred Reporting Items for Systematic reviews and Meta-Analyses 2020 (PRISMA 2020) statement (24) and the recommendations outlined in the Cochrane Handbook (25), ensuring standardized procedures for study selection, data extraction, statistical analysis, and result interpretation. The study protocol was prospectively registered in the International Prospective Register of Systematic Reviews (PROSPERO) (CRD420251132513).

### Study inclusion and exclusion criteria

The inclusion criteria were established using the PICOS framework.

Population (P): Adult patients (≥18 years) with acute IS who underwent EVT (e.g., mechanical thrombectomy, intra-arterial thrombolysis).

Intervention/exposure (I): Measurement of GAR, defined as FPG divided by HbA1c, assessed at admission or within 24 h of admission. Patients with a high GAR were considered the exposure, with the cutoffs for defining high GAR consistent with the values used in the original studies.

Comparator (C): Patients with a low GAR at baseline.

Outcomes (O): Functional outcomes, defined as a modified Rankin Scale (mRS) score of 3–6 at 90 days, compared between patients in the high vs. the low GAR categories.

Study design (S): Longitudinal observational studies, such as cohort studies (prospective or retrospective), nested case–control studies, or *post hoc* analysis of randomized controlled trials (RCTs).

Studies were excluded if they (1) did not involve patients undergoing EVT for IS; (2) lacked assessment of GAR (fasting glucose/HbA1c) as the exposure of interest or analyzed GAR only as a continuous variable; (3) did not report functional outcomes at follow-up; (4) provided insufficient data to calculate effect estimates or lacked a comparator group; (5) were case reports, reviews, editorials, meta-analyses, or animal studies; or (6) were duplicate publications or involved overlapping populations with the same dataset. For studies with overlapping populations, the analysis included the one with the largest sample size. Although we initially planned to exclude studies reporting only continuous GAR due to the potentially insurmountable and error-prone conversion of *β* coefficients or hazard ratios into odds ratios (ORs) for pooling, no study was excluded for this reason during literature screening.

### Database search

We performed a comprehensive literature search in PubMed, Embase, Web of Science, Wanfang, and Chinese National Knowledge Infrastructure (CNKI) to identify eligible studies. The search strategy combined terms related to: (1) glycemic parameters, including “relative hyperglycemia,” “acute-to-chronic glycemic ratio,” “glucose-to-glycated hemoglobin,” “glucose-to-HbA1c,” “stress hyperglycemia ratio,” “SHR,” and “GAR”; (2) disease terms such as “ischemic stroke,” “stroke,” “cerebral infarction,” and “cerebrovascular infarction”; (3) interventions, including “endovascular therapy,” “mechanical thrombectomy,” “intra-arterial thrombolysis,” “neurointervention,” “endovascular treatment,” and related procedures; and (4) outcomes and study design descriptors, such as “mortality,” “survival,” “prognosis,” “functional outcome,” “cohort,” “prospective,” “retrospective,” and “follow-up.” The search was limited to human studies and full-text articles published in English or Chinese in peer-reviewed journals. Reference lists of relevant articles and reviews were also screened to identify additional studies. The search period extended from database inception to 29 May 2025. The full search strategy for each database is shown in Supplementary File S1.

### Study quality evaluation

Two authors independently conducted the literature search, study selection, quality assessment, and data extraction, with disagreements resolved through discussion with the corresponding author. Study quality was evaluated using the Newcastle–Ottawa Scale (NOS) (26), which assesses selection, control of confounders, and outcome evaluation, with total scores ranging from 1 to 9 and scores ≥ 8 indicating high quality.

### Data extraction

Data extracted for analysis included study characteristics (author, year, country, and design), patient information [sample size, diagnosis, mean age, sex distribution, baseline National Institutes of Health Stroke Scale (NIHSS) score, proportion of diabetic patients, and type of EVT], exposure details (timing of GAR measurement, methods and cutoff values used to define high GAR), incidence of poor functional outcomes at 90 days, and covariates adjusted for in the regression analyses examining the association between GAR and 90-day outcomes.

### Statistical analysis

We used ORs with 95% confidence intervals (CIs) to evaluate the association between GAR and 90-day functional outcomes in IS patients after EVT. ORs and standard errors were directly obtained or derived from 95% CIs or *p*-values and then log-transformed to stabilize variance and normalize the data (25). When multiple models were available, we selected the OR with the most comprehensive adjustment. Heterogeneity was assessed using the Cochrane Q test and the I^2^ statistic (27), with *p* < 0.10 indicating significant heterogeneity and I^2^ values < 25, 25–75, and > 75 representing low, moderate, and high heterogeneity, respectively. A random-effects model was applied to account for between-study variation (25). Sensitivity analyses were performed by sequentially excluding each study to test the robustness of the findings (25). Univariate meta-regression was conducted to explore whether study-level factors, such as sample size, mean age, sex distribution, baseline NIHSS, proportion of diabetic patients, GAR cutoff methods, incidence of poor outcomes, and study quality scores, influenced the pooled estimates (25). Pre-specified subgroup analyses were carried out according to patient age, sex distribution, baseline NIHSS, diabetic status, methods for defining GAR cutoffs, cutoff values, and NOS scores, using median values of continuous variables for stratification. Publication bias was examined through visual inspection of funnel plots and Egger’s test (28). All statistical analyses were performed using RevMan (Version 5.3; Cochrane Collaboration, Oxford, UK) and Stata (Version 17.0; Stata Corporation, College Station, TX, USA). A *p* < 0.05 indicates statistical significance. The certainty of evidence for the primary outcome was assessed using the Grading of Recommendations Assessment, Development and Evaluation (GRADE) framework, evaluating five domains (risk of bias, inconsistency, indirectness, imprecision, and publication bias) (25). A Summary of Findings table was generated to present the certainty ratings and reasons for downgrading or upgrading accordingly.

## Results

### Study selection

**Figure 1:**
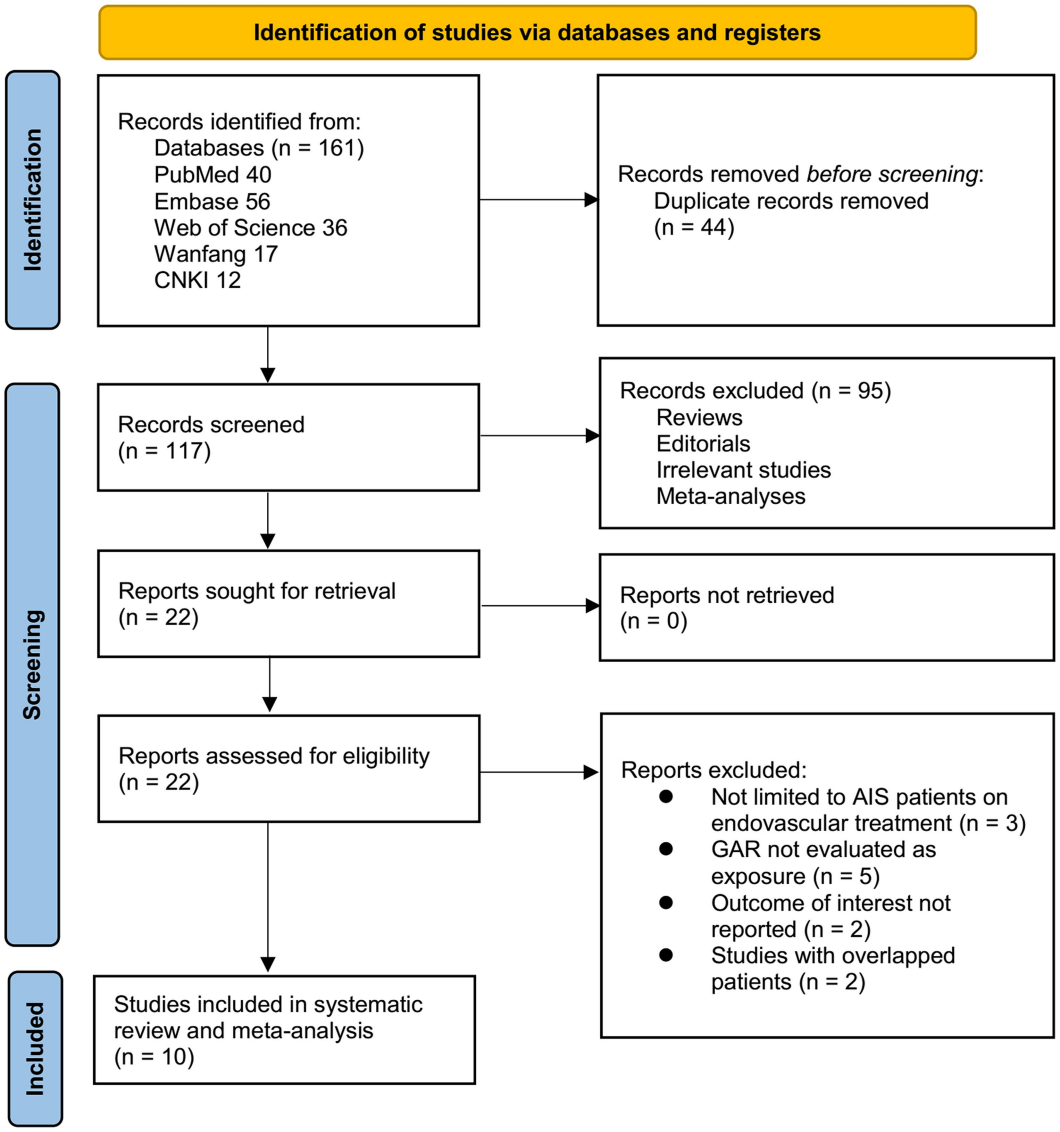
Flowchart of database search and study inclusion.

The study selection process is summarized in Figure 1. A total of 161 records were initially retrieved from five databases. After removing 44 duplicates, 117 articles underwent title and abstract screening, with 95 excluded for not meeting the inclusion criteria. The full texts of the remaining 22 articles were reviewed independently by two authors, and 12 were excluded for various reasons (Figure 1). Ultimately, 10 studies were included in the quantitative analysis (14–23).

### Study characteristics

Table 1 summarizes the key characteristics of the 12 datasets derived from 10 studies included in this meta-analysis (14–23). Two studies (16, 17) reported outcomes separately for patients with and without diabetes, and these were analyzed as independent datasets, making 12 datasets available for the meta-analysis. The studies were published between 2021 and 2025 and conducted across Italy, the United Kingdom, Japan, and China. All were retrospective cohort studies and together included 3,878 patients with acute IS treated with EVT. Sample sizes ranged from 79 to 691 patients, with mean ages spanning 62.0 to 75.5 years, and the proportion of male patients ranging from 45.4 to 66.5%. Baseline stroke severity, measured by the NIHSS, varied between studies, with mean values from 12.6 to 16.5. GAR was consistently measured within 24 h of hospitalization or symptom onset, but detailed information on whether the glucose sample was strictly fasting, or on the exact timing relative to the EVT procedure, was generally not reported. The cutoff values for defining high vs. low GAR varied: most studies used the highest vs. lowest quartile (Q4: Q1) (14, 16, 18, 20, 21, 23), while others applied tertile-based cutoffs (T3: T1) (17, 22) or receiver operating characteristic (ROC) curve analysis (15, 19). The cutoff values for defining a high GAR varied from 1.07 to 1.50. Poor functional outcome was uniformly defined as an mRS score of 3–6 at 90 days, with incidence rates ranging from 34.0 to 69.1%. Overall, 2,125 (54.8%) patients had a poor functional outcome at 90 days. Multivariate analysis was performed in all studies. Covariate adjustment varied across studies, with most models including age, sex, baseline NIHSS, vascular risk factors (e.g., hypertension, diabetes, atrial fibrillation), pre-stroke mRS, imaging parameters (e.g., Alberta Stroke Program Early CT Score, collateral status), and procedural factors such as time to reperfusion.

**Table 1:** Characteristics of the included studies.

Study	Design	Country	Number of patients with stroke	Mean age (years)	Male (%)	Mean NIHSS at admission	DM (%)	Details of EVT	Timing of GAR evaluation	Methods for defining GAR cutoff	Cutoff value of high GAR (mmol/L per %)	Number of patients with poor functional outcome	Incidence (%) of poor functional outcome	Variables adjusted
Merlino et al. (14)	R	Italy	204	74.4	49	16.5	10.3	MT (40.3% direct MT, 59.3% MT + intravenous alteplase)	Within 24 h of hospitalization	Q4: Q1	1.22	122	59.8	Age, history of DM, ASPECTS score, baseline NIHSS score, pre-stroke mRS, time from symptom onset to MT, door-to-groin time, procedure duration
Zhang et al. (19)	R	China	408	64.8	65	14.5	34.1	MT for anterior circulation large-vessel occlusion	Within 24 h of hospitalization	Q4: Q1	1.49	282	69.1	Age, sex, previous TIA/stroke, history of hypertension, smoking, hemoglobin, hs-CRP, SBP, NIHSS score at admission, pre-stroke mRS, and IV tPA administration
Dai et al. (15)	R	China	559	70	63.9	13.9	25.8	MT (with Solitaire and/or Catalyst6 devices)	Within 24 h of hospitalization	ROC curve analysis	1.07	275	49.2	Age, baseline NIHSS, NIHSS at 24 h, puncture-to-recanalization time, pre-procedure ASITN/SIR grade, and successful recanalization
Duan et al. (16) NDM	R	China	346	66.4	51.8	15.3	0	MT and/or stent implantation	Within 24 h of symptom onset	Q4: Q1	1.33	147	42.5	Age, CHD, AF, NIHSS at admission, any ICH
Duan et al. (16) DM	R	China	230	68	52.6	14.5	100	MT and/or stent implantation	Within 2 4 h of symptom onset	Q4: Q1	1.50	122	53.0	Age, hypertension, hyperlipidemia, previous stroke, NIHSS at admission, any ICH, respiratory failure
Zhang et al. (20)	R	China	159	69.8	61.6	12.6	35.8	MT (stent retriever, aspiration, or a combination)	Within 24 h of symptom onset	ROC curve analysis	1.21	54	34.0	Age, baseline NIHSS, ASITN/SIR collateral grade, and symptomatic ICH
Wang and Fan (17) NDM	R	China	130	65.9	56.2	14.4	0	MT with complete recanalization	Within 24 h of hospitalization	T3: T1	1.36	67	51.5	Age, hemoglobin, TG, HDL, admission NIHSS, admission SBP, ASITN/SIR collateral score, ASPECTS, and atherosclerotic stroke etiology
Wang and Fan (17) DM	R	China	79	69.9	46.8	14.3	100	MT with complete recanalization	Within 24 h of hospitalization	T3: T1	1.41	49	62.0	Age, AF, admission DBP, and onset to reperfusion time
Yang et al. (21)	R	China	553	62	66.5	15	24.6	MT	Within 24 h of hospitalization	Q4: Q1	1.39	318	57.5	Age, sex, SBP, NIHSS score at admission, pre-stroke mRS, CVD, previous TIA/stroke, smoking, hemoglobin, and serum creatinine
Merlino et al. (20)	R	Italy and UK	691	75.5	45.4	16.3	14	MT	Within 24 h of hospitalization	Q4: Q1	1.38	403	58.3	Age, sex, hypertension, AF, anticoagulant Use, NIHSS at admission, ASPECTS, site of Occlusion (Tandem vs. MCA), number of retrieval attempts >3, procedure length, and alteplase use before MT
Gao et al. (22)	R	China	415	72	61.4	14	29.2	MT (stent retriever, aspiration, or a combination)	Within 24 h of hospitalization	T3: T1	1.18	246	59.3	Age, sex, hypertension, DM, AF, pre-stroke mRS score, admission SBP, baseline NIHSS, BUN, eGFR, successful recanalization, door-to-puncture time
Tsuji et al. (23)	R	Japan	104	73.1	66.3	15.4	24	MT	Within 24 h of hospitalization	Q4: Q1	1.25	40	38.5	Age, SBP, DWI-ASPECTS score, number of passes, puncture-to-reperfusion time

AF, atrial fibrillation; ASITN/SIR, American Society of Interventional and Therapeutic Neuroradiology/Society of Interventional Radiology; ASPECTS, Alberta Stroke Program Early CT Score; BUN, blood urea nitrogen; CVD, cardiovascular disease; DBP, diastolic blood pressure; DM, diabetes mellitus; DWI, diffusion-weighted imaging; eGFR, estimated glomerular filtration rate; EVT, endovascular treatment; GAR, glucose-to-HbA1c ratio; HDL, high-density lipoprotein; hs-CRP, high-sensitivity C-reactive protein; ICH, intracerebral hemorrhage; IV tPA, intravenous tissue plasminogen activator; MCA, middle cerebral artery; mRS, modified Rankin Scale; MT, mechanical thrombectomy; NIHSS, National Institutes of Health Stroke Scale; Q4: Q1, highest quartile vs. lowest quartile; ROC, receiver operating characteristic; SBP, systolic blood pressure; TIA, transient ischemic attack; TG, triglyceride; T3: T1, highest quartile vs. lowest tertile.

### Study quality assessment

Table 2 presents the quality assessment of the included datasets using the NOS. Overall, methodological quality was moderate to high, with total scores ranging from 8 to 9 out of a maximum of 9. Six datasets achieved the maximum score of 9 (14, 17, 20–22), while the remaining six scored 8 points (15, 16, 18, 19, 23). Lower scores were mainly attributed to the limited representativeness of the exposed cohort or slightly incomplete follow-up. All datasets demonstrated robust ascertainment of exposure, appropriate selection of non-exposed cohorts, and reliable outcome assessment, supporting the validity of the data synthesized in this meta-analysis.

**Table 2:** Study quality evaluation via the Newcastle–Ottawa Scale.

Study	Representativeness of the exposed cohort	Selection of the non-exposed cohort	Ascertainment of exposure	Outcome not present at baseline	Control for age	Control for other confounding factors	Assessment of outcome	Enough long follow-up duration	Adequacy of follow-up of the cohort	Total
Merlino et al. (14)	1	1	1	1	1	1	1	1	1	9
Zhang et al. (19)	1	1	1	1	1	1	1	1	0	8
Dai et al. (15)	0	1	1	1	1	1	1	1	1	8
Duan et al. (16) NDM	0	1	1	1	1	1	1	1	1	8
Duan et al. (16) DM	0	1	1	1	1	1	1	1	1	8
Zhang et al. (20)	0	1	1	1	1	1	1	1	1	8
Wang and Fan (17) NDM	1	1	1	1	1	1	1	1	1	9
Wang and Fan (17) DM	1	1	1	1	1	1	1	1	1	9
Yang et al. (21)	1	1	1	1	1	1	1	1	1	9
Merlino et al. (20)	1	1	1	1	1	1	1	1	1	9
Gao et al. (22)	1	1	1	1	1	1	1	1	1	9
Tsuji et al. (23)	1	1	1	1	1	1	0	1	1	8

### Meta-analysis and sensitivity analysis results

**Figure 2:**
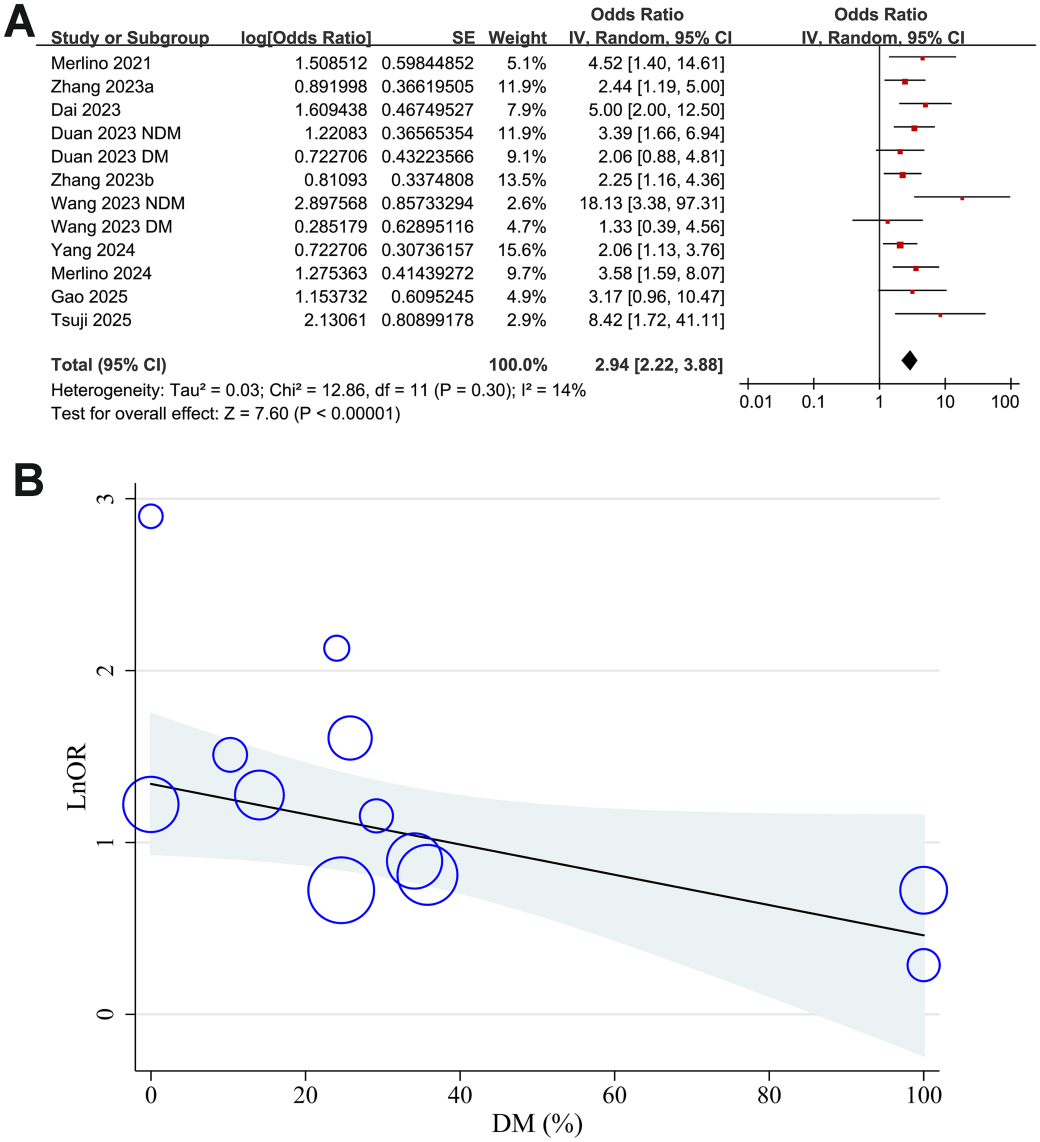
Forest plots and meta-regression analysis examining the impact of diabetes on the association between GAR and 90-day poor functional outcome in patients with IS after EVT. **(A)** Overall meta-analysis; **(B)** correlation between the proportion of patients with diabetes and the pooled OR.

Mild heterogeneity was observed among the included studies evaluating the association between GAR and 90-day functional outcome of IS patients after EVT (*p* for Cochrane Q test = 0.30; I^2^ = 14%). The pooled results of the 12 datasets (14–23) with a random-effects model showed that overall, a high GAR at baseline was associated with a higher risk of poor functional outcome at 90 days after EVT (OR: 2.94, 95% CI: 2.22 to 3.88, *p* < 0.001; Figure 2A). Sensitivity analyses were performed by removing one dataset at a time, and the results remained stable (OR: 2.76 to 3.13, all *p* < 0.05).

### Meta-regression analysis results

Results of the univariate meta-regression analysis are presented in Table 3. The proportion of patients with diabetes in each study showed a negative, though not statistically significant, correlation with the pooled OR for the association between GAR and poor functional outcome (coefficient = −0.0088, *p* = 0.08; Table 3 and Figure 2B). Notably, variation in diabetic status across datasets appeared to fully account for the observed heterogeneity (I^2^ residual = 0%). In contrast, other study-level factors, including sample size, mean age, proportion of men, baseline NIHSS, GAR cutoff values, incidence of poor functional outcome, and study quality scores, did not demonstrate any meaningful association with the pooled results.

**Table 3:** Results of univariate meta-regression analysis.

Variables	OR for the association between high GAR and the risk of a 90-day poor functional outcome			
	Coefficient	95% CI	*p-*values	I^2^ residual (%)
Sample size	0.000095	−0.00164 to 0.00183	0.91	22.1
Mean age (years)	0.0040	−0.0323 to 0.1122	0.25	10.5
Men (%)	−0.0073	−0.0499 to 0.0354	0.71	21.1
Baseline NIHSS	0.11	−0.17 to 0.39	0.40	16.3
DM (%)	−0.0088	−0.0188 to 0.0012	0.08	0
Cutoff of GAR (mmol/L)	−1.51	−3.86 to 0.85	0.19	6.5
Incidence of poor functional outcome (%)	−0.0064	−0.0359 to 0.0232	0.64	20.5
NOS	−0.014	−0.669 to 0.642	0.96	22.2

CI, confidence interval; DM, diabetes mellitus; GAR, glucose-to-HbA1c ratio; NIHSS, National Institutes of Health Stroke Scale; NOS, Newcastle–Ottawa Scale; OR, odds ratio.

### Subgroup analysis results

**Figure 3:**
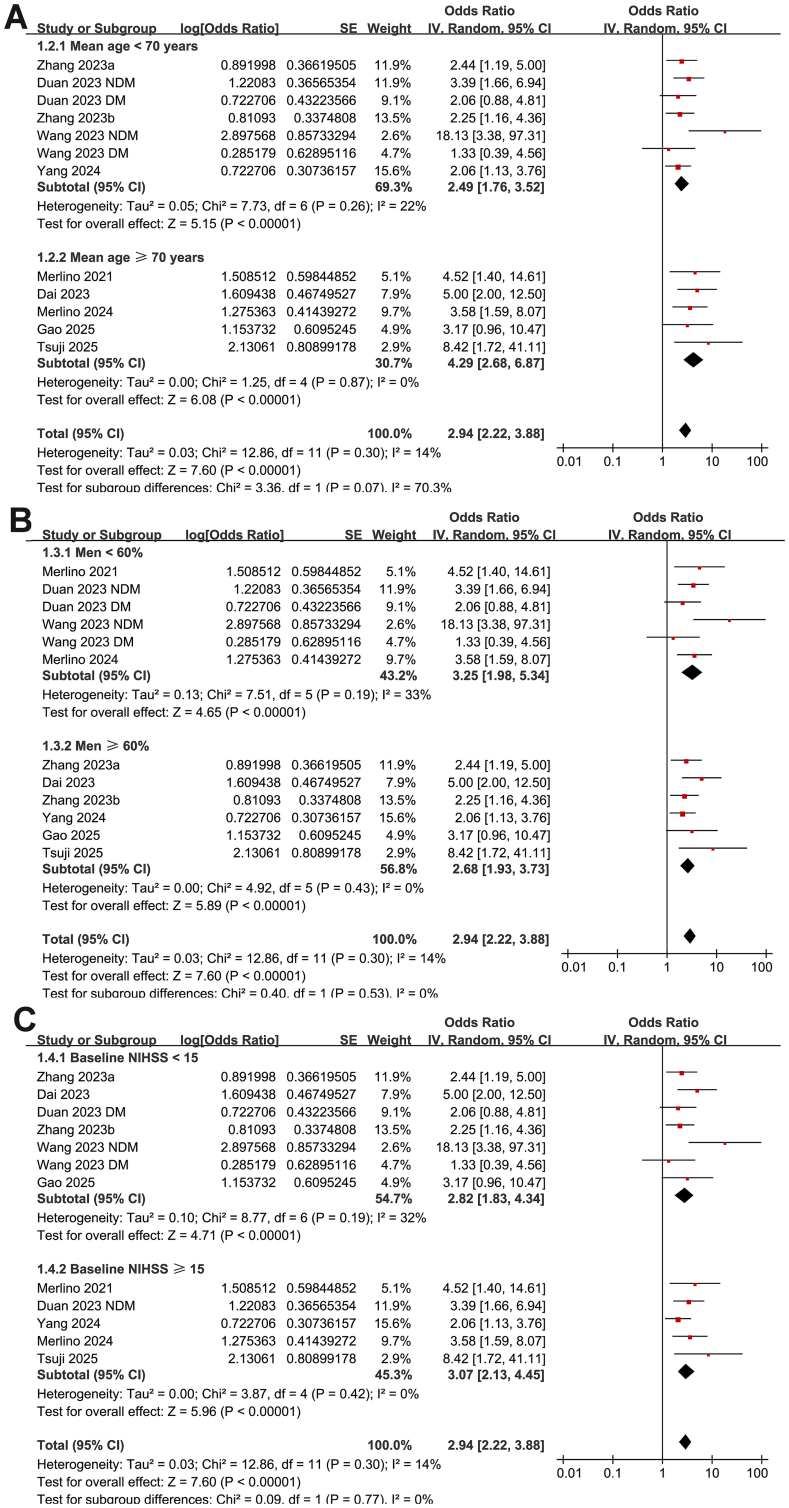
Forest plots for the subgroup analyses of the association between GAR and 90-day poor functional outcome in patients with IS after EVT. **(A)** Subgroup analysis according to mean ages of the patients; **(B)** subgroup analysis according to the proportion of men; **(C)** subgroup analysis according to the mean NIHSS at admission.

**Figure 4:**
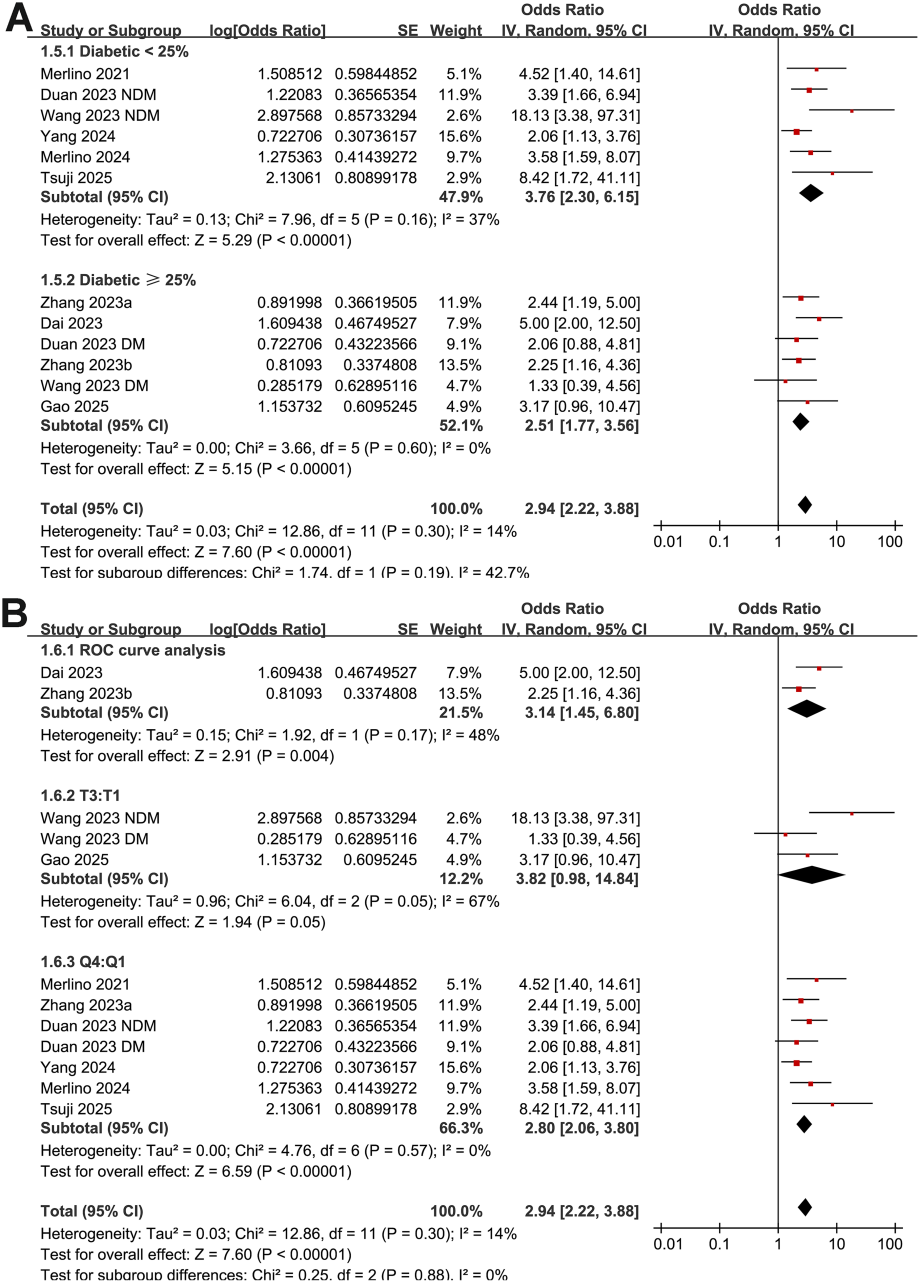
Forest plots for the subgroup analyses of the association between GAR and 90-day poor functional outcome in patients with IS after EVT. **(A)** Subgroup analysis according to the proportion of patients with diabetes; **(B)** subgroup analysis according to the methods for defining cutoffs of GAR.

**Figure 5:**
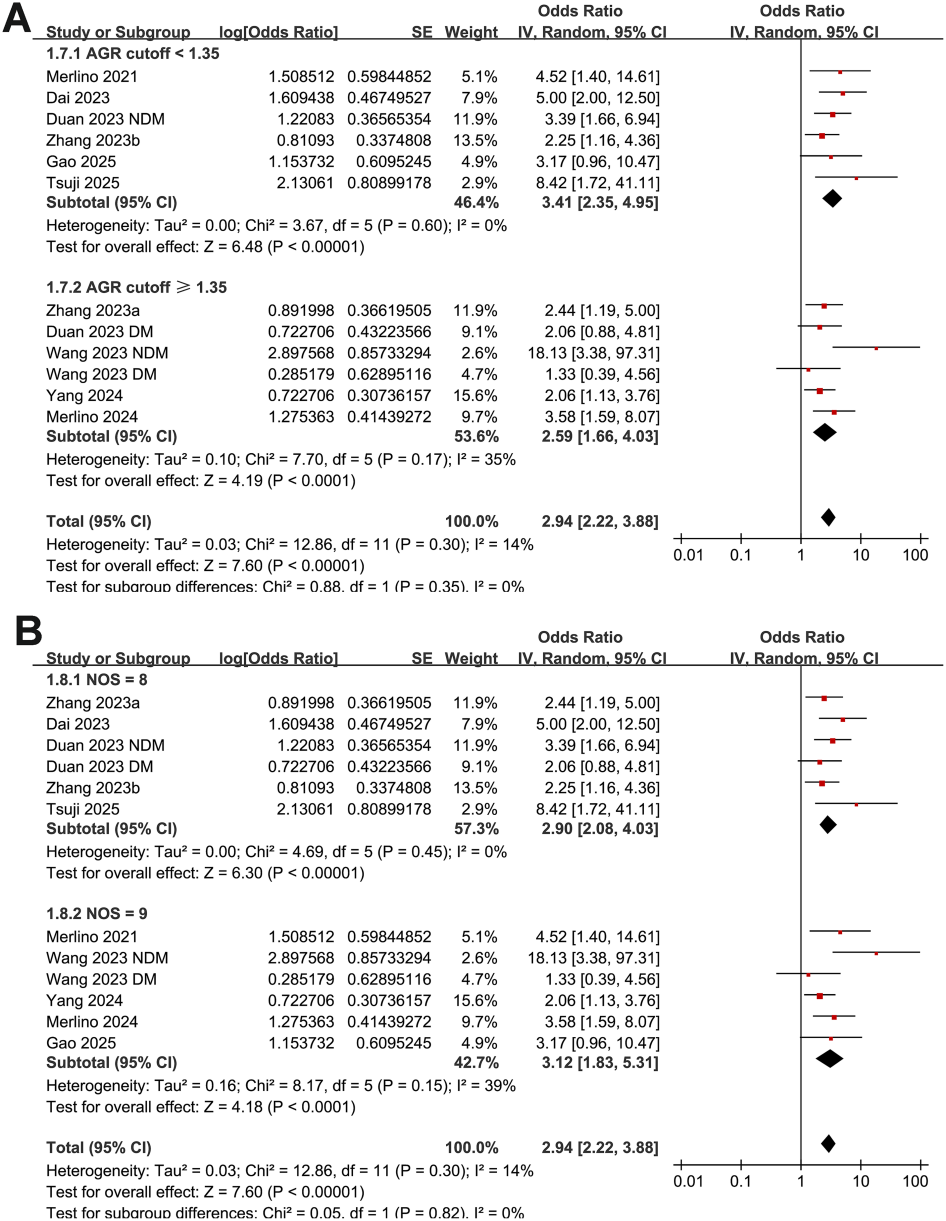
Forest plots for the subgroup analyses of the association between GAR and 90-day poor functional outcome in patients with IS after EVT. **(A)** Subgroup analysis according to the cutoff values of GAR; **(B)** subgroup analysis according to the NOS scores.

Further subgroup analyses showed that the association between GAR and the increased risk of poor functional outcome at 90 days after EVT remained consistent across multiple study-level factors. Similar results were observed in studies with mean patient age < or ≥ 70 years (OR: 2.49 vs. 4.29, *p* for subgroup difference = 0.07; Figure 3A), proportion of men < or ≥ 60% (OR: 3.25 vs. 2.68, *p* = 0.53; Figure 3B), and mean baseline NIHSS < or ≥ 15 (OR: 2.82 vs. 3.07, *p* = 0.77; Figure 3C). Comparable findings were also noted for studies with a proportion of diabetic patients < or ≥ 25% (OR: 3.76 vs. 2.51, *p* = 0.19; Figure 4A), for those defining high GAR by quartiles, tertiles, or ROC analysis (OR: 2.80, 3.82, and 3.14, *p* = 0.88; Figure 4B), for studies with cutoff values for high GAR < or ≥ 1.35 (OR: 3.41 vs. 2.59, *p* = 0.35; Figure 5A), and for studies with NOS scores of 8 or 9 (OR: 2.90 vs. 3.12, *p* = 0.82; Figure 5B).

### Publication bias test

**Figure 6:**
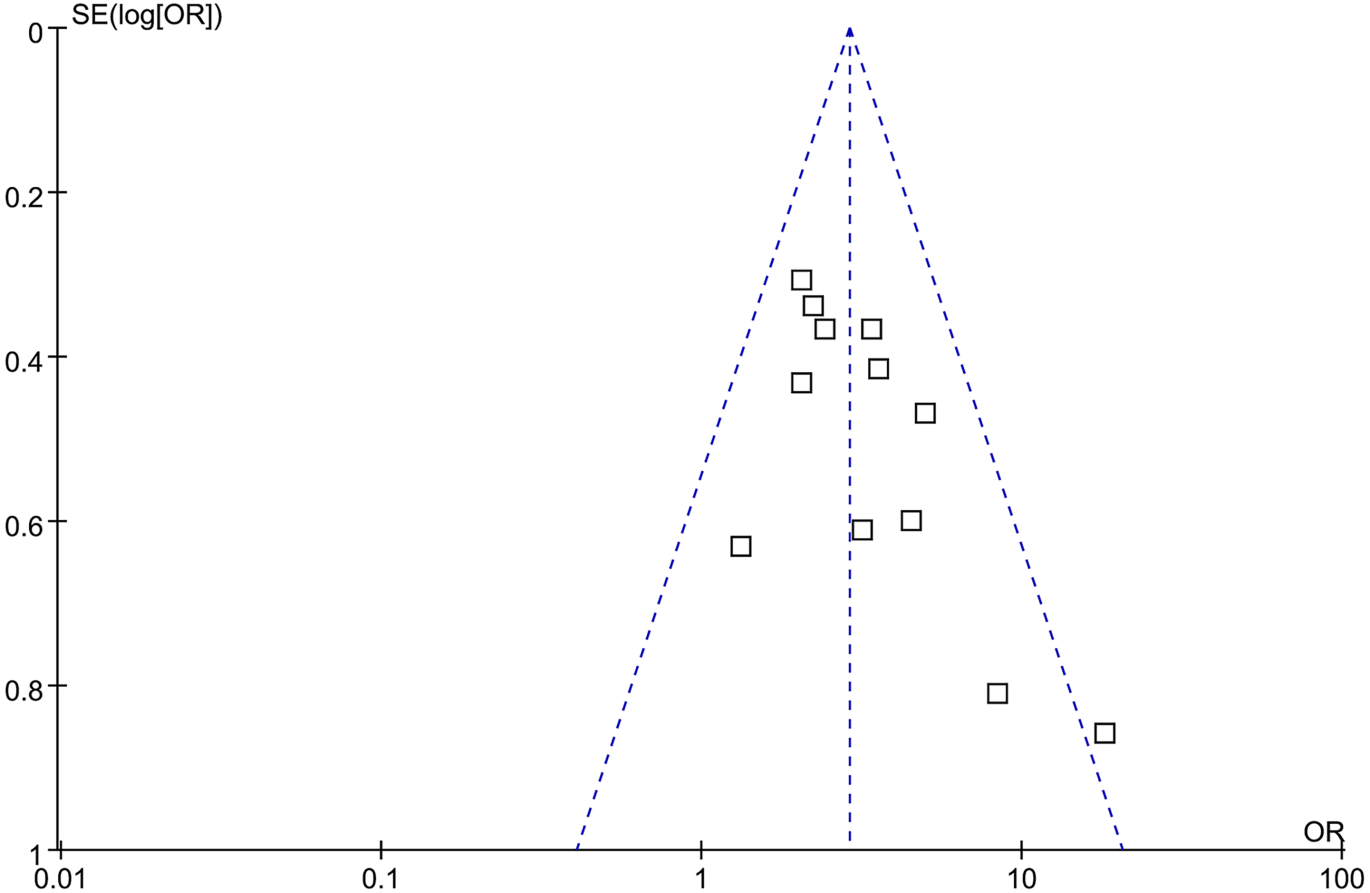
Funnel plots for estimating the potential publication bias underlying the meta-analysis of the association between GAR and 90-day poor functional outcome in patients with IS after EVT.

Funnel plots assessing the association between GAR and the risk of 90-day poor functional outcome are presented in Figure 6. The plots appeared symmetrical, indicating a low likelihood of publication bias, which was further supported by Egger’s test (*p* = 0.38).

### Certainty of evidence

According to the GRADE assessment (Supplementary Table S1), the certainty of evidence for the association between high GAR and poor 90-day functional outcome after EVT was rated as moderate, mainly because all included studies were retrospective cohorts.

## Discussion

This meta-analysis provides the most comprehensive evidence to date linking an elevated GAR, a marker of stress hyperglycemia adjusted for chronic glycemia, with poor 90-day functional outcomes in IS patients undergoing EVT. By including 12 datasets from 10 studies with multivariate-adjusted effect estimates, our findings build upon prior research on stress hyperglycemia in acute stroke but extend them by specifically focusing on EVT-treated patients and by differentiating GAR from other commonly used indices such as admission glucose/HbA1c or admission glucose/estimated average glucose ratios. This distinction is clinically meaningful because these indices, while all adjusted for chronic glycemia, differ in their susceptibility to prandial effects and short-term glycemic fluctuations, factors that may influence prognostic accuracy. Our study demonstrates that GAR, particularly when derived from fasting glucose values, provides robust and independent prognostic information across diverse patient subgroups, with results remaining stable in sensitivity and meta-regression analyses.

Several biological mechanisms may underlie the association between elevated GAR and adverse functional outcomes after EVT. Stress hyperglycemia reflects a neuroendocrine and inflammatory response triggered by acute cerebral ischemia, involving activation of the hypothalamic–pituitary–adrenal axis, catecholamine release, and cytokine-driven insulin resistance (29, 30). This acute hyperglycemic surge promotes oxidative stress, endothelial dysfunction, and blood–brain barrier disruption, thereby exacerbating cerebral edema and increasing susceptibility to hemorrhagic transformation (31, 32). Hyperglycemia also amplifies excitotoxicity by enhancing glutamate release and intracellular calcium accumulation, impairing mitochondrial function, and accelerating neuronal apoptosis (33, 34). Furthermore, elevated glucose levels inhibit fibrinolysis through increased plasminogen activator inhibitor-1 expression, which may worsen microvascular reperfusion despite successful large-vessel recanalization (35). Importantly, GAR incorporates fasting blood glucose (FBG) and HbA1c, thus accounting for premorbid glycemia and distinguishing transient stress hyperglycemia from prandial effect and chronic hyperglycemia due to diabetes (36, 37). This is clinically relevant because transient glucose surges appear more deleterious than chronic hyperglycemia alone, possibly owing to the abrupt metabolic and oxidative shifts imposed on previously normoglycemic tissues (38). Indeed, oscillating glucose levels have been shown to induce greater endothelial injury and inflammatory responses than sustained hyperglycemia, providing a plausible explanation for the observed association between high GAR and poor neurological recovery (39, 40).

Subgroup analyses in our study revealed that the association between elevated GAR and poor outcomes persisted across strata defined by age, sex, baseline stroke severity, diabetes prevalence, GAR cutoff methods, and study quality, supporting the robustness and generalizability of our findings. Interestingly, meta-regression showed a trend toward an influence of diabetes prevalence on the modest heterogeneity observed (*p* = 0.08), with weaker associations reported in cohorts with higher proportions of diabetic patients. Although not statistically significant, this finding aligns with biological plausibility: patients with long-standing diabetes may develop adaptive mechanisms to chronic hyperglycemia, such as upregulation of antioxidant defenses or ischemic preconditioning, mitigating the additional harm imposed by acute glucose surges (41). In contrast, stress hyperglycemia in previously normoglycemic individuals likely represents a novel metabolic insult superimposed upon acute cerebral ischemia, provoking greater oxidative and inflammatory injury (42). Similar observations were reported in previous studies, where stress hyperglycemia predicted poor outcomes more strongly in non-diabetic than diabetic patients, reinforcing the need for future individual patient data (IPD) meta-analyses to delineate such interactions more precisely (43). In addition, sensitivity analyses further confirmed the stability of our findings, as sequential exclusion of individual studies did not materially alter pooled estimates. Moreover, no significant publication bias was detected using Egger’s test, although the possibility of small-study effects cannot be entirely excluded given the modest number of available studies. Taken together, these results indicate that the adverse prognostic impact of elevated GAR after EVT is unlikely to be driven by a single outlier study or by selective reporting bias, lending credibility to the observed associations.

Our findings also complement and extend two previous meta-analyses that investigated stress hyperglycemia indices in acute ischemic stroke but did not differentiate between admission glucose/HbA1c, fasting glucose/HbA1c, and admission glucose/estimated average glucose ratios, nor did they focus specifically on EVT-treated patients (36, 37). Both studies confirmed that elevated stress hyperglycemia indices were associated with worse functional outcomes, higher mortality, and increased risk of hemorrhagic complications after ischemic stroke (36, 37). However, by pooling heterogeneous indices and patient populations, including those managed medically or with intravenous thrombolysis alone, prior analyses could not identify which parameter or clinical context conferred the strongest prognostic utility (36, 37). Our meta-analysis addresses this gap by restricting inclusion to EVT-treated patients and by analyzing GAR separately from other indices, thereby demonstrating that GAR, particularly when based on fasting glucose, may offer superior and more consistent prognostic value.

The present study also has several methodological strengths. We conducted a comprehensive and up-to-date literature search across multiple databases and included only cohort studies with multivariate-adjusted risk estimates. All included studies evaluated 90-day functional outcomes using the mRS, ensuring consistency in endpoint definition. Furthermore, prespecified subgroup, sensitivity, and meta-regression analyses were performed to explore heterogeneity and assess robustness. At the same time, all studies enrolled consecutive EVT-treated patients, enhancing clinical relevance to contemporary stroke practice. Nonetheless, several limitations warrant cautious interpretation. First, all included studies were retrospective cohorts, introducing risks of selection bias, residual confounding, and incomplete data capture (44). Second, substantial heterogeneity in patient comorbidities, stroke severity, EVT techniques (e.g., device type, anesthesia modality, and reperfusion time), and operator experience could not be fully explored due to a lack of IPD. Third, definitions and cutoff values for high GAR varied across studies, although subgroup analyses stratified by cutoff determination methods yielded consistent results. Fourth, despite multivariate adjustment in individual studies, unmeasured confounders such as peri-procedural glucose management, nutritional status, infection, or withdrawal-of-care decisions might still influence outcomes. Another limitation is the lack of standardized timing and fasting status for glucose measurement across studies. While most reports specified that GAR was calculated within 24 h of admission or symptom onset, few clarified whether patients were fasted or whether samples were taken before, during, or after the EVT procedure. As EVT is often performed urgently, GAR values may have been influenced by peri-procedural stress responses, potentially contributing to between-study variability. Finally, the observational design precludes causal inference; whether stress hyperglycemia directly worsens neurological recovery or merely reflects overall illness severity remains unresolved.

From a clinical perspective, GAR offers several advantages as a prognostic biomarker. It is inexpensive, widely available, and easily calculated from routine laboratory tests, requiring no specialized equipment or delayed assays. By incorporating chronic glycemia, GAR improves upon absolute glucose values in distinguishing transient stress hyperglycemia from pre-existing diabetes, thereby refining early risk stratification after EVT. Patients with markedly elevated GAR might benefit from closer hemodynamic and metabolic monitoring, early initiation of neuroprotective strategies, or enrollment into future trials testing intensive glucose control in the hyper-acute phase of IS. However, given the observational nature and methodological limitations of current evidence, routine clinical implementation of GAR-based interventions should await confirmation from large-scale prospective studies and randomized controlled trials.

## Conclusion

In conclusion, this meta-analysis demonstrates that elevated GAR is independently associated with an increased risk of poor 90-day functional outcomes in IS patients undergoing EVT. By distinguishing GAR from other stress hyperglycemia indices and focusing specifically on EVT-treated populations, our findings provide novel insights into the prognostic significance of acute-on-chronic glycemic imbalance in IS. Future multi-center prospective studies with standardized GAR assessment, IPD-level analyses, and interventional designs are needed to validate these findings, establish optimal cutoff values, and determine whether GAR-guided metabolic management can improve neurological recovery after EVT.

## Data Availability

The original contributions presented in the study are included in the article/Supplementary material, further inquiries can be directed to the corresponding author.
