# Supplementary material for: Fasting blood glucose-to-glycated hemoglobin ratio and functional outcomes in patients with ischemic stroke following endovascular treatment—a meta-analysis
Source: Front Neurol. 2025 Dec 4;16:1692103. doi: 10.3389/fneur.2025.1692103 (PMC12715375; doi:10.3389/fneur.2025.1692103)
Supplement: Supplementary file 1 [file Table_1.DOCX]

**Supplementary File 1** Detailed search strategy for each database

**PubMed**

("relative hyperglycemia"[tiab] OR "acute-to-chronic glycemic ratio"[tiab] OR "glucose-to-glycated hemoglobin"[tiab] OR "glucose to glycated hemoglobin"[tiab] OR "glucose-to-HbA1c"[tiab] OR "glucose to HbA1c"[tiab] OR "stress hyperglycemia ratio"[tiab] OR "stress-hyperglycaemia ratio"[tiab] OR SHR[tiab] OR GAR[tiab]) AND ("Stroke"[Mesh] OR "Brain Ischemia"[Mesh] OR "Cerebral Infarction"[Mesh] OR "ischemic stroke"[tiab] OR stroke[tiab] OR "cerebral infarction"[tiab] OR "brain infarction"[tiab] OR "cerebrovascular infarction"[tiab]) AND ("Thrombectomy"[Mesh] OR "Endovascular Procedures"[Mesh] OR thrombectomy[tiab] OR "mechanical thrombectomy"[tiab] OR "endovascular therapy"[tiab] OR "endovascular treatment"[tiab] OR "endovascular thrombectomy"[tiab] OR neurointervention*[tiab] OR "intra-arterial thrombolysis"[tiab] OR intra-arterial[tiab] OR intraarterial[tiab] OR stent*[tiab]) AND ("Mortality"[Mesh] OR "Prognosis"[Mesh] OR "Treatment Outcome"[Mesh] OR "Survival Rate"[Mesh] OR mortality[tiab] OR survival[tiab] OR prognosis[tiab] OR outcome*[tiab] OR "functional outcome"[tiab] OR "clinical outcome"[tiab] OR cohort[tiab] OR longitudinal[tiab] OR prospective[tiab] OR retrospective[tiab] OR prospectively[tiab] OR retrospectively[tiab] OR "follow-up"[tiab] OR followed[tiab])

**Embase**

('relative hyperglycemia':ti,ab,kw OR 'acute to chronic glycemic ratio':ti,ab,kw OR 'glucose-to-glycated hemoglobin':ti,ab,kw OR 'glucose to glycated hemoglobin':ti,ab,kw OR 'glucose-to-HbA1c':ti,ab,kw OR 'glucose to HbA1c':ti,ab,kw OR 'stress hyperglycemia ratio':ti,ab,kw OR 'stress-hyperglycaemia ratio':ti,ab,kw OR SHR:ti,ab,kw OR GAR:ti,ab,kw) AND ('ischemic stroke'/exp OR 'cerebral infarction'/exp OR 'brain ischemia'/exp OR 'ischemic stroke':ti,ab,kw OR stroke:ti,ab,kw OR 'cerebral infarction':ti,ab,kw OR 'brain infarction':ti,ab,kw OR 'cerebrovascular infarction':ti,ab,kw) AND ('thrombectomy'/exp OR 'endovascular therapy'/exp OR 'endovascular procedure'/exp OR 'intraarterial drug administration'/exp OR 'stent'/exp OR 'mechanical thrombectomy':ti,ab,kw OR 'endovascular therapy':ti,ab,kw OR 'endovascular treatment':ti,ab,kw OR 'endovascular thrombectomy':ti,ab,kw OR neurointervention*:ti,ab,kw OR 'intra-arterial thrombolysis':ti,ab,kw OR 'intra-arterial':ti,ab,kw OR intraarterial:ti,ab,kw OR stent*:ti,ab,kw) AND ( 'mortality'/exp OR 'prognosis'/exp OR 'treatment outcome'/exp OR 'survival'/exp OR mortality:ti,ab,kw OR survival:ti,ab,kw OR prognosis:ti,ab,kw OR outcome*:ti,ab,kw OR 'functional outcome':ti,ab,kw OR 'clinical outcome':ti,ab,kw OR cohort:ti,ab,kw OR longitudinal:ti,ab,kw OR prospective:ti,ab,kw OR retrospective:ti,ab,kw OR prospectively:ti,ab,kw OR retrospectively:ti,ab,kw OR 'follow-up':ti,ab,kw OR followed:ti,ab,kw)

**Web of Science**

TS=(("relative hyperglycemia" OR "acute-to-chronic glycemic ratio" OR "glucose-to-glycated hemoglobin" OR "glucose to glycated hemoglobin" OR "glucose-to-HbA1c" OR "glucose to HbA1c" OR "stress hyperglycemia ratio" OR "stress-hyperglycaemia ratio" OR SHR OR GAR) AND ("ischemic stroke" OR stroke OR "cerebral infarction" OR "brain infarction" OR "cerebrovascular infarction") AND ("endovascular therapy" OR "mechanical thrombectomy" OR thrombectomy OR "intra-arterial thrombolysis" OR "endovascular thrombectomy" OR neurointervention* OR "endovascular treatment" OR endovascular OR stent* OR "intra-arterial" OR intraarterial) AND (mortality OR survival OR prognosis OR outcome* OR "functional outcome" OR "clinical outcome" OR cohort OR longitudinal OR prospective OR retrospective OR prospectively OR retrospectively OR "follow-up" OR followed))

**Wanfang**

((主题:相对高血糖 OR 主题:急慢性血糖比 OR 主题:血糖-糖化血红蛋白 OR 主题:血糖与糖化血红蛋白 OR主题:血糖-HbA1c OR 主题:血糖与HbA1c OR 主题:应激性高血糖比值 OR 主题:压力性高血糖比值 OR主题:SHR OR 主题:GAR)) AND (主题:缺血性卒中 OR 主题:缺血性脑卒中 OR 主题:脑梗死 OR 主题:脑梗塞 OR 主题:脑梗) AND (主题:血管内治疗 OR 主题:机械取栓 OR 主题:取栓 OR 主题:动脉内溶栓 OR 主题:血管内取栓 OR 主题:神经介入 OR 主题:支架 OR 主题:动脉内) AND (主题:死亡 OR 主题:死亡率 OR 主题:生存 OR 主题:预后 OR 主题:功能结局 OR 主题:临床结局 OR 主题:随访 OR 主题:队列 OR 主题:前瞻性 OR 主题:回顾性))

**CNKI (Chinese National Knowledge Infrastructure)**

(主题=('相对高血糖' OR '急慢性血糖比' OR '血糖-糖化血红蛋白' OR '血糖与糖化血红蛋白' OR '血糖-HbA1c' OR '血糖与HbA1c' OR '应激性高血糖比值' OR '压力性高血糖比值' OR 'SHR' OR 'GAR')) AND (主题=('缺血性卒中' OR '缺血性脑卒中' OR '脑梗死' OR '脑梗塞' OR '脑梗')) AND (主题=('血管内治疗' OR '机械取栓' OR '取栓' OR '动脉内溶栓' OR '血管内取栓' OR '神经介入' OR '支架' OR '动脉内')) AND (主题=('死亡' OR '死亡率' OR '生存' OR '预后' OR '功能结局' OR '临床结局' OR '随访' OR '队列' OR '前瞻性' OR '回顾性'))
