# Supplementary material for: Fasting blood glucose-to-glycated hemoglobin ratio and functional outcomes in patients with ischemic stroke following endovascular treatment—a meta-analysis
Source: Front Neurol. 2025 Dec 4;16:1692103. doi: 10.3389/fneur.2025.1692103 (PMC12715375; doi:10.3389/fneur.2025.1692103)
Supplement: Supplementary file 2 [file Table_2.DOCX]

**Supplementary Table 1** GRADE evidence profile: Association between high GAR and poor 90-day functional outcome after EVT in ischemic stroke

The certainty of evidence was assessed using the GRADE (Grading of Recommendations Assessment, Development and Evaluation) framework. This approach evaluates five domains: risk of bias, inconsistency, indirectness, imprecision, and publication bias. Evidence from observational studies begins at “Low” certainty and may be downgraded or upgraded depending on study limitations.

| Outcome | No. of studies (datasets) | Study design | Risk of bias | Inconsistency | Indirectness | Imprecision | Publication bias / Overall certainty |
| --- | --- | --- | --- | --- | --- | --- | --- |
| Association between high GAR and poor 90-day functional outcome (mRS 3–6) after EVT | 10 studies (12 datasets) | Retrospective cohort | Serious (all observational and retrospective; residual confounding) | Not serious (low heterogeneity, I² = 14%) | Not serious (directly relevant population, exposure, and outcome) | Not serious (large effect, CI narrow; OR = 2.94, 95% CI 2.22–3.88) | Not serious (funnel plot symmetric; Egger’s test *p* = 0.38) Overall certainty: Moderate (downgraded one level for study limitations) |

Notes:

• Risk of bias: Downgraded one level because all included studies were retrospective cohorts, with potential selection bias and unmeasured confounding.

• Inconsistency: Not downgraded because heterogeneity was low (I² = 14%).

• Indirectness: Not downgraded because the studies directly evaluated EVT-treated ischemic stroke patients, GAR measured at baseline, and the prespecified outcome of 90-day poor functional status (mRS 3–6).

• Imprecision: Not downgraded because the effect estimate was robust with a narrow confidence interval.

• Publication bias: Not downgraded as funnel plots appeared symmetrical and Egger’s test (*p* = 0.38) suggested low risk.

• Overall certainty: Rated as Moderate because the evidence started at “Low” for observational design, was upgraded one level for a large consistent effect across subgroups, but then downgraded back one level for residual risk of bias.
